# Numerical Integration of Stochastic Differential Equations: The Heun Algorithm Revisited and the Itô-Stratonovich Calculus

**DOI:** 10.3390/e27090910

**Published:** 2025-08-28

**Authors:** Riccardo Mannella

**Affiliations:** Dipartimento di Fisica, Università di Pisa, 56126 Pisa, Italy; riccardo.mannella@unipi.it

**Keywords:** stochastic differential equations (SDEs), numerical integration, Heun’s method, strong convergence, stochastic processes, Itô Stratonovich calculus

## Abstract

The widely used Heun algorithm for the numerical integration of stochastic differential equations (SDEs) is critically re-examined. We discuss and evaluate several alternative implementations, motivated by the fact that the standard Heun scheme is constructed from a low-order integrator. The convergence, stability, and equilibrium properties of these alternatives are assessed through extensive numerical simulations. Our results confirm that the standard Heun scheme remains a benchmark integration algorithm for SDEs due to its robust performance. As a byproduct of this analysis, we also disprove a previous claim in the literature regarding the strong convergence of the Heun scheme.

## 1. Introduction

Stochastic Differential Equations (SDEs) are an indispensable tool for modeling complex systems across a vast range of scientific disciplines, including physics, chemistry, biology, and finance [1,2]. These equations provide a powerful framework for describing systems that are subject to random fluctuations, or “noise,” which is often an intrinsic feature of the underlying dynamics. Unlike Ordinary Differential Equations (ODEs), which have deterministic solutions, the solution to an SDE is itself a stochastic process, representing a collection of possible trajectories.

A central challenge in working with SDEs is that most non-trivial cases cannot be solved analytically. Consequently, their analysis relies heavily on numerical integration schemes to approximate solutions [3]. The development of robust and efficient numerical algorithms is therefore of paramount importance. An ideal scheme must balance several competing factors: it should be accurate, converging to the true solution as the integration time step decreases; it must be stable, avoiding divergence even for large time steps or stiff problems; and it should be computationally efficient.

Among the many algorithms developed for this purpose, the Heun scheme has emerged as a widely used and reliable method. As a stochastic extension of the classic Runge–Kutta predictor-corrector method, it is known for its favorable balance of simplicity, stability, and accuracy. Its structure naturally handles the Itō-Stratonovich dilemma, converging to the Stratonovich interpretation of the SDE, which is often preferred in physical modeling where the noise represents a smoothed, physical process rather than a purely mathematical construct [4,5].

Despite its widespread use, the standard Heun scheme is built upon the Euler–Maruyama method, a scheme with a relatively low order of strong convergence. This fact motivates a critical re-examination: Could the performance of the Heun method be improved by incorporating higher-order building blocks? How do its stability and accuracy compare to other, more complex Taylor-based or Runge–Kutta-type schemes?

This paper addresses these questions through a systematic investigation of the Heun algorithm and its variants. We critically evaluate several alternative implementations, comparing their performance in terms of strong convergence, numerical stability, and their ability to reproduce the correct long-time equilibrium distributions of a non-trivial SDE. Our findings confirm that the standard Heun scheme remains a benchmark algorithm for its robustness and reliability. As a byproduct of this analysis, we also re-evaluate and ultimately disprove a previous claim in the literature regarding the strong convergence order of the Heun scheme.

## 2. The Heun Algorithm and Itô-Stratonovich Calculus

The Heun algorithm for the numerical integration of Stochastic Differential Equations (SDEs) is a stochastic extension of an integrator first introduced by Heun in 1900 [6]. That paper addressed the then-nascent field of Runge–Kutta schemes for integrating Ordinary Differential Equations (ODEs) [7].

Given the ODE:

(1) $$\dot{x}=f\left(x,t\right),$$

the Heun scheme calculates the next step, $x_{n+1}\equiv x\left(t+h\right)$, from the current step, $x_{n}\equiv x\left(t\right)$, as follows:

(2) $$\begin{array}{cc} \tilde{x} & =x_{n}+hf\left(x_{n},t\right)\\ \overset{\approx }{x} & =x_{n}+hf\left(\tilde{x},t+h\right)\\ x_{n+1} & =\frac{1}{2}\left(\tilde{x}+\overset{\approx }{x}\right)=x_{n}+\frac{h}{2}\left[f\left(x_{n},t\right)+f\left(\tilde{x},t+h\right)\right]. \end{array}$$

Equation (2) shows that the Heun scheme is a *predictor-corrector* method. It first *predicts* a value with an explicit Euler step and then *corrects* it by averaging this explicit step with an implicit Euler step.

One of the earliest papers to mention a stochastic version of the Heun algorithm is [8], where this name was used to refer to a stochastic Runge–Kutta scheme derived in [9]. Given the one-dimensional SDE:

(3) dx=f(x,t)dt+g(x,t)dW,

where *W* is a standard Wiener process, the well-known Euler–Maruyama (in the following, Euler) scheme in the *Itô sense* is:

(4) $$x_{n+1}=x_{n}+hf\left(x_{n},t\right)+g\left(x_{n},t\right)Z_{1},$$

where $Z_{1}=Z_{1}\left(h\right)\equiv \int_{t}^{t+h}dW=\sqrt{h}Y_{1}$, with $Y_{1}∼N\left(0,1\right)$, is a Gaussian random variable with zero mean and variance *h*. In [8], the Heun scheme for SDEs was written as:

(5) $$x_{n+1}=x_{n}+\frac{h}{2}\left[f\left(x_{n},t\right)+f\left(\tilde{x},t+h\right)\right]+\frac{1}{2}\left[g\left(x_{n},t\right)+g\left(\tilde{x},t+h\right)\right]Z_{1},$$

where the predictor step is:

(6) $$\tilde{x}=x_{n}+hf\left(x_{n},t\right)+g\left(x_{n},t\right)Z_{1},$$

and the same random variable $Z_{1}$ is used in both stages. Much like its deterministic counterpart, the stochastic Heun scheme can be seen as the average of an explicit and an implicit stochastic Euler step.

It is well known (see, for example, [8,9,10]) that the stochastic evolution described by Equation (5) converges to the evolution of Equation (3) interpreted in the *Stratonovich* sense. This is equivalent to the following SDE in the Itô sense:

(7) $$dx=\left(f\left(x,t\right)+\frac{1}{2}g\left(x,t\right)\frac{dg(x,t)}{dx}\right)dt+g\left(x,t\right)dW.$$

It was claimed in [9] that the Heun scheme is a scheme with a strong convergence order $O(h^{3/2})$ (See Section 4.1 for a definition of strong convergence. Also, we will loosely use $O(h^{\alpha })$ and $o(h^{\alpha })$ when terms proportional to $h^{\alpha }$ are kept or discarded, respectively). Furthermore, in [11], it was demonstrated that for a potential V(x) bounded from below (i.e., $\inf_{x}V\left(x\right)>-\infty$), with $f\left(x\right)=-V^{\prime }\left(x\right)$ and $g\left(x\right)=\sqrt{2D}$, the scheme in Equation (5) yields the correct equilibrium distribution for *x* up to order $o(h^{2})$.

In the following sections, we will consider only single-step algorithms with a time step *h*. For simplicity, we will use the notation $x\left(h\right)\equiv x_{n+1}$ and $x\left(0\right)\equiv x_{n}$. When there is no ambiguity, we will use shorthand such as f≡f(x,t) and $g^{\prime }\equiv \frac{dg(x,t)}{dx}$. Subscripts will be added when necessary to avoid confusion (e.g., $f_{0}\equiv f\left(x_{0},t_{0}\right)$).

Although it may seem unsurprising that the Heun scheme yields a Stratonovich evolution—given that its elementary step averages quantities evaluated at the beginning and end of the time interval, which mirrors the definition of Stratonovich calculus [5]—several questions naturally arise:

- The Heun scheme produces a Stratonovich evolution by combining two Euler schemes that use the Itô prescription. Should one instead use Euler schemes derived from the Stratonovich prescription?
- The standard stochastic Euler scheme has a strong convergence order of $O(h^{1/2})$ [3], unlike the deterministic Euler scheme. Would it be beneficial to replace it in the Heun scheme with an integrator that has a strong convergence order of O(h)?
- How does the Heun scheme compare with other Taylor-based higher-order schemes?

To address these points, some background is necessary (see also [5]).

### 2.1. The Stochastic Euler Scheme and Stochastic Calculus

An elementary O(h) scheme for Equation (3), obtained via a Taylor expansion around $x_{0}$, is given by (see, for example, [12]):

(8) $$x\left(h\right)=x\left(0\right)+g_{0}Z_{1}+hf_{0}+g_{0}g_{0}^{\prime }\int_{0}^{h}W\left(s\right)\;dW\left(s\right).$$

If Itô calculus is used, the integral is $\int_{0}^{h}W\left(s\right)\;dW\left(s\right)=\frac{1}{2}\left(Z_{1}^{2}-h\right)$, which leads to the Milstein scheme (see Equation (11) below). If this term is approximated as zero, the standard Euler scheme (Equation (4)) is recovered. If Stratonovich calculus is used, the integral is $\int_{0}^{h}W\left(s\right)\circ dW\left(s\right)=\frac{1}{2}Z_{1}^{2}$, which yields the Euler–Stratonovich scheme:

(9) $$x\left(h\right)=x\left(0\right)+g_{0}Z_{1}+hf_{0}+\frac{1}{2}g_{0}g_{0}^{\prime }Z_{1}^{2}.$$

The Heun algorithm requires an Euler step involving the final point, x(h). For Itô calculus, this step corresponds to Equation (4), replacing $x_{n}$ with $x_{n+1}$ in the arguments of f(x) and g(x). In contrast, when using Stratonovich calculus, expanding around x(h) results in:

(10) $$x\left(h\right)=x\left(0\right)+g_{h}Z_{1}+hf_{h}-\frac{1}{2}g_{h}g_{h}^{\prime }Z_{1}^{2}.$$

### 2.2. Strong Approximation O(h): The Milstein Scheme

A strong approximation O(h) for integrating Equation (3) was proposed by Milstein [13]:

(11) $$x\left(h\right)=x\left(0\right)+g_{0}Z_{1}+hf_{0}+\frac{1}{2}g_{0}g_{0}^{\prime }\left(Z_{1}^{2}-h\right).$$

Comparing this to Equation (7), the Milstein scheme corresponds to applying the Euler–Stratonovich scheme (Equation (9)) to the following SDE (The symbol ∘ indicates that this SDE is to be interpreted in the Stratonovich sense):

(12) $$dx=\left(f\left(x,t\right)-\frac{1}{2}g\left(x,t\right)\frac{dg(x,t)}{dx}\right)dt+g\left(x,t\right)\circ dW.$$

### 2.3. Higher-Order Taylor-Based Schemes

A Taylor-based scheme of order $O(h^{2})$ using Itô calculus was derived in [14], and a revised version using Stratonovich calculus was developed in [12]. Even higher-order Taylor-based schemes were derived in [3], leveraging a chain rule for higher-order multiple stochastic integrals.

As shown both analytically and numerically in [11], care must be taken when deriving higher-order schemes. Specifically, if the terms containing stochastic integrals are computed to a certain order, the deterministic terms must be kept at the same order. One should not include higher-order deterministic terms, even if they are easy to derive (The deterministic terms are often simpler to compute, which might tempt one to include them regardless of the order achieved in the stochastic part). Doing so can result in a scheme that performs worse than one where all terms—both stochastic and deterministic—are consistently maintained at the same order of accuracy.

In the following, we will use the results of [12,15], which are reproduced here for convenience:

(13) $$\begin{array}{ccccc} x(h)=\; & x\left(0\right)+g_{0}Z_{1}+hf_{0}+\frac{1}{2}g_{0}g_{0}^{\prime }Z_{1}^{2} & & O\left(h^{1/2}\right)+O\left(h\right) & \\ & +\left(g_{0}f_{0}^{\prime }-f_{0}g_{0}^{\prime }\right)Z_{2}+hf_{0}g_{0}^{\prime }Z_{1}+\frac{1}{3!}g_{0}\left(g_{0}^{\prime }g_{0}^{\prime }+g_{0}^{\prime \prime }g_{0}\right)Z_{1}^{3} & & O(h^{3/2}) & \\ & +\frac{h^{2}}{2}f_{0}f_{0}^{\prime }+\frac{1}{2}f_{0}^{\prime }g_{0}^{\prime }g_{0}\left(Z_{1}Z_{2}-Z_{3}\right)+\frac{1}{2}f_{0}^{\prime \prime }g_{0}g_{0}\left(Z_{1}Z_{2}-Z_{3}\right) & & O(h^{2}) & \\ & +g_{0}^{\prime }\left(g_{0}f_{0}^{\prime }-f_{0}g_{0}^{\prime }\right)Z_{3}+g_{0}^{\prime }g_{0}^{\prime }f_{0}\frac{1}{2}\left(hZ_{1}^{2}-\frac{1}{2}Z_{1}Z_{2}+\frac{1}{2}Z_{3}\right) & & O(h^{2}) & \\ & +\frac{1}{4!}g_{0}\left(g_{0}^{2}g_{0}^{\prime \prime \prime }+g_{0}^{\prime }{\left(g_{0}g_{0}^{\prime }\right)}^{\prime }\right)Z_{1}^{4}+\frac{h}{4}g_{0}^{\prime \prime }g_{0}f_{0}\left(Z_{1}^{2}-\frac{1}{2}\left(Z_{1}Z_{2}-Z_{3}\right)\right) & & O(h^{2}) & \end{array}$$

where two additional stochastic integrals appear: $Z_{2}=Z_{2}\left(h\right)\equiv \int_{0}^{h}Z_{1}\left(s\right)ds=\int_{0}^{h}\left(\int_{0}^{s}dW\right)ds$ and $Z_{3}=Z_{3}\left(h\right)\equiv \int_{0}^{h}Z_{2}\left(s\right)dW\left(s\right)$. A suitable representation for $Z_{2}$ is easily found, as it is Gaussian [12,14]:

(14) $$Z_{2}=Z_{2}\left(h\right)=h\left(\frac{Z_{1}}{2}+\frac{\sqrt{h}}{2\sqrt{3}}Y_{2}\right),$$

where $Y_{2}∼N\left(0,1\right)$ is independent of $Y_{1}$. Finding a representation for $Z_{3}$ is much harder, as a number of constraints must be satisfied. Considering the lowest orders in *h*, the following moments are found:

$$\begin{array}{c} \langle Z_{3}\left(h\right)\rangle =0,\;\langle {\left[Z_{3}\left(h\right)\right]}^{2}\rangle =\frac{h^{4}}{12}\;\langle Z_{1}\left(h\right)Z_{3}\left(h\right)\rangle =0,\;\langle Z_{2}\left(h\right)Z_{3}\left(h\right)\rangle =0\\ \langle {\left[Z_{1}\left(h\right)\right]}^{2}Z_{3}\left(h\right)\rangle =\frac{1}{4}h^{3},\;\langle Z_{1}\left(h\right)Z_{2}\left(h\right)Z_{3}\left(h\right)\rangle =\frac{h^{4}}{12}\;\langle {\left[Z_{2}\left(h\right)\right]}^{2}Z_{3}\left(h\right)\rangle =\frac{h^{5}}{15}, \end{array}$$

where 〈…〉 denotes an average over stochastic realizations. The number of conditions to satisfy exceeds the number of unknown quantities, so an arbitrary choice must be made. In [12], the representation

(15) $$Z_{3}\left(h\right)=\frac{h^{2}}{6}\left\{Y_{1}^{2}-h+Y_{3}\right\}$$

was used (A misprint in Ref. [12] has been corrected here.), where $Y_{3}∼N\left(0,1\right)$ is independent of $Y_{1}$ and $Y_{2}$. In preparation for this manuscript, an alternative representation was derived:

(16) $$Z_{3}=Z_{1}Z_{2}-\frac{h^{2}}{2}\left(1+\frac{Y_{3}}{3}\right).$$

## 3. Algorithms and Dynamical System

The numerical schemes studied here are typically used to integrate SDEs to derive equilibrium or quasi-equilibrium properties. These include calculating the mean first passage time to a boundary, deriving reaction rate constants, and determining the long-time equilibrium distribution.

It is therefore relevant to test these approaches using a non-trivial SDE with a known equilibrium distribution. Verifying agreement with the full distribution is a more stringent test than merely checking the behavior of a few moments or cumulants. This focus on long-time accuracy is at odds with the typical analysis of integration schemes, which often investigates the behavior of stochastic trajectories over short time scales.

However, it is possible in some cases to connect these two regimes. Using the formalism of [16], one can infer the equilibrium distribution generated by a given numerical scheme from its short-time properties [11,17]. This connection demonstrates that schemes with more accurate short-time behavior also produce numerical equilibrium distributions that are closer to the theoretical one.

The dynamical system used for the test is one of the simplest non-trivial models:

(17) $$dx=-x\left(1+x^{2}\right)dt+\sqrt{2D}\left(1+x^{2}\right)dW=f\left(x\right)dt+g\left(x\right)dW.$$

This model has the equilibrium distribution

(18) $$P_{eq}\left(x\right)=\frac{N}{{\left(1+x^{2}\right)}^{1+\alpha +n}},$$

where α=1/(2D), *n* is 0 for Stratonovich calculus and 1 for Itô calculus, and $N=\Gamma \left(1+n+\alpha \right)/(\Gamma \left(3/2+\alpha \right)\sqrt{\pi })$ is a normalization constant. It is noteworthy that the presence of a power-law tail makes the comparison with the numerical schemes particularly interesting because the different schemes must be able to integrate correctly even when the variable *x* becomes large.

The algorithms tested are as follows, where the labels will be used for identification below:

**Euler:** The standard Euler scheme, Equation (4).
**Heun:** The standard Heun scheme, Equation (5).
**Stra:** The Euler–Stratonovich scheme, Equation (9).
**Miln:** A modified Heun scheme where the Milstein algorithm (Equation (11)) is used as the basic block for both the predictor and corrector steps: in the corrector step, f(x) and g(x) are evaluated at the *x* found in the predictor step.
**HeSt:** A modified Heun scheme using the Euler–Stratonovich scheme of Equation (9) for the predictor step and Equation (10) for the corrector step.
**HePC:** An iterated Heun scheme. First, Equation (5) is used to obtain a tentative final point. This point is then re-inserted into Equation (5) in place of $\tilde{x}$. In our simulations, this loop was iterated four times.
**Mil-:** A modified Heun scheme similar to **Miln**, where for the corrector step the − sign in front of the term $\frac{1}{2}gg^{\prime }$ is used (this resembles what is done in Equation (10) for the Euler–Stratonovich scheme)
**T3/2:** The scheme of Equation (13), including terms up to the ones marked $O(h^{3/2})$
**HPC-:** An iterated Heun scheme similar to **HePC** but using Stratonovich schemes Equations (9) and (10) rather than Euler.
**CUP1:** The scheme of Equation (13) using Equation (15)
**CUP2:** The scheme of Equation (13) using Equation (16)
**RK:** The efficient RK scheme of [18].

## 4. Numerical Results and Discussion

All codes were written in Fortran 90 and parallelized using the MPICH [19] library on machines with 24, 30, or 40 cores. The random number generator used was RAN2 [20] from Numerical Recipes.

### 4.1. Convergence

We assessed the convergence rate of the different schemes following the approach of [21], where further details can be found. The core idea is that if $x_{f}$ is the final point of a trajectory integrated with a time step *h* from an initial point $x_{0}$, and $x_{limit}$ is the “true” final point obtained in the limit of h→0 starting from the same $x_{0}$, the expected error satisfies $E|x_{f}-x_{limit}|\leq Ah^{\alpha }$ for an algorithm with a strong convergence of order α.

In the case studied in [21], $x_{limit}$ was found by solving the SDE analytically. However, since the exact solution to the SDE in Equation (17) is not known, we adopted the following procedure for each algorithm:

- A random initial point was chosen from the Stratonovich equilibrium distribution Equation (18).
- The trajectory was integrated from the initial point up to time t=1.0 with a very small time step $h_{s}=1/2^{17}\approx 7.63\times 10^{-6}$, storing the random variates generated at each step. This trajectory serves as the “reference” trajectory, and its final point $x_{ref}$ was stored.
- The integration was repeated from the same initial point but with a larger integration time step $h_{n}=2^{n}h_{s}$, for 1≤n≤13, and the final point $x_{f}\left(h_{n}\right)$ was stored. To generate the noise for these larger time steps, the random increments from the reference trajectory were summed. For example, if the noise increments for the reference trajectory from *t* to $t+h_{s}$ and from $t+h_{s}$ to $t+2h_{s}$ were $w_{1}$ and $w_{2}$, respectively, and the noise increment for a trajectory with time step $h_{1}=2h_{s}$ from *t* to $t+2h_{s}$ was taken as $w_{1}+w_{2}$. This procedure was extended straightforwardly for cases requiring multiple random terms.
- The absolute error $|x_{f}\left(h_{n}\right)-x_{ref}|$ was computed and stored.
- The procedure was repeated for $N=4\times 10^{6}$ trajectories, and the average error, $E[|x_{f}\left(h_{n}\right)-x_{ref}|]$, was computed.
- Finally, a fit of the average error versus *h* was performed using MINUIT2 [22] to determine the parameters *A* and α.

For integration time steps where $h\gg h_{s}$, it is reasonable to consider $x_{ref}$ a proxy for the true value $x_{limit}$. The fit was performed using simulation data in the range $10^{-4}\leq h\leq 10^{-2}$.

**Figure 1:**
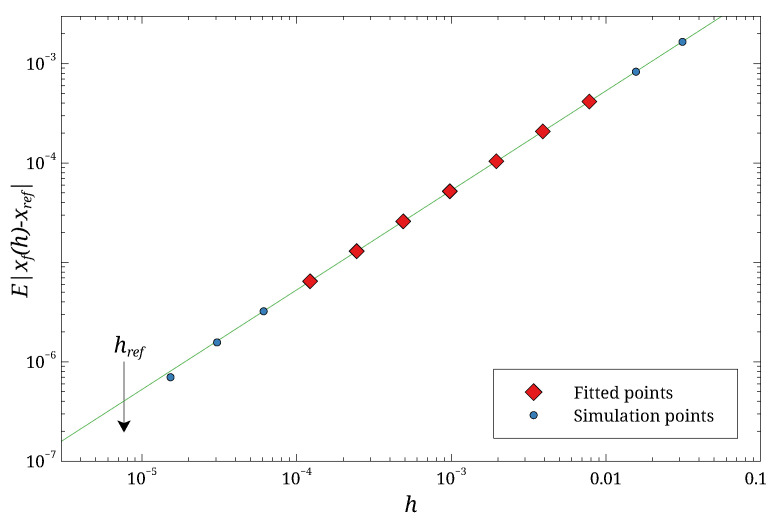
Typical data used for the convergence fit, showing $E|x_{f}\left(h\right)-x_{ref}|$ vs. *h*. The data shown are for the Heun algorithm. The solid line is the fit to the model $f\left(h\right)=Ah^{\alpha }$. The symbols are the results of simulations: the red diamonds were used for the fit, while the light blue circles were excluded. The arrow labeled $h_{ref}$ marks the *h* used for the reference trajectory. The statistical error due to the finite number of averages is smaller than the symbol size.

A typical case is shown in Figure 1. Note that the values of *h* used in the fit are significantly larger than $h_{ref}$. The results of the fit are summarized in Table 1. The points marked with light blue circles were excluded from the fit but are shown to verify the expected linear relationship on a log-log plot within the fitting range. To validate our method, we first tested the standard Euler scheme. As expected, the fit yields α=0.53±0.02, which is consistent with the theoretical convergence order of 0.5 for the Euler scheme.

**Table 1:** Table summarizing strong convergence and stability for each scheme. The convergence order is in the form $\propto Ah^{\alpha }$, and the table shows the value of the parameters *A* and α found in the fit; in the simulations for the convergence, D=0.05. The column “Stability” reports the number of red dots appearing on each plate of Figure 2.

Algorithm	*A*	α	Stability
Euler	$\left(1.8\pm 0.3\right)\times 10^{-2}$	0.53±0.02	
Heun	$\left(5.4\pm 0.5\right)\times 10^{-2}$	1.00±0.01	191
Stra	$\left(9.6\pm 0.9\right)\times 10^{-2}$	1.01±0.01	115
Miln	$\left(1.8\pm 0.2\right)\times 10^{-2}$	0.52±0.01	91
HeSt	$\left(5.9\pm 0.6\right)\times 10^{-2}$	1.00±0.01	172
HePC	$\left(5.4\pm 0.5\right)\times 10^{-2}$	1.00±0.01	169
Mil-	$\left(5.6\pm 0.5\right)\times 10^{-2}$	1.00±0.01	165
HPC-	$\left(6.2\pm 0.6\right)\times 10^{-2}$	1.00±0.01	132
T3/2	$\left(9.2\pm 0.9\right)\times 10^{-2}$	1.01±0.01	134
CUP1	$\left(7.7\pm 0.7\right)\times 10^{-2}$	1.01±0.01	117
CUP2	$\left(7.7\pm 0.7\right)\times 10^{-2}$	1.01±0.01	117
RK	$\left(2.4\pm 0.2\right)\times 10^{-2}$	0.85±0.01	135

**Figure 2:**
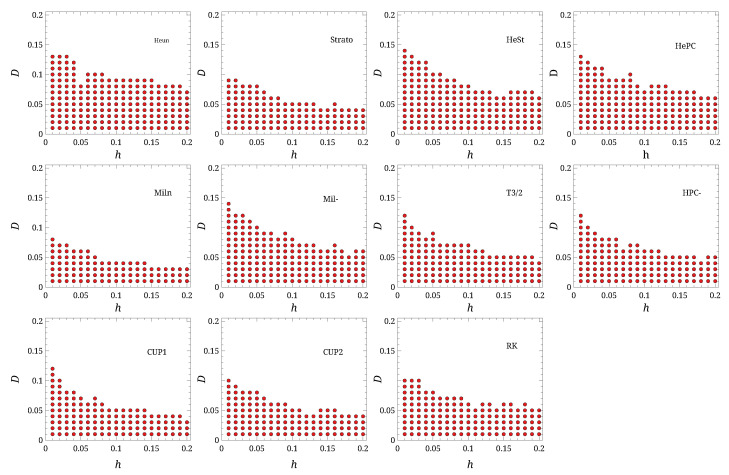
Stability regions for the different algorithms, as function of *D* and *h*. The red dots mark the parameters for which the given scheme did not blow up.

Most schemes show strong convergence of order O(h). The simple Stratonovich scheme (**Stra**, Equation (9)) also has O(h) convergence. This may seem surprising given that the Euler scheme is only $O(h^{1/2})$, but it is consistent with the fact that the Stratonovich scheme includes terms up to O(h) from the Taylor expansion in Equation (13).

Interestingly, the Heun scheme variant that uses the Milstein algorithm (**Miln**) as its building block achieves only $O(h^{1/2})$ strong convergence. However, O(h) convergence is recovered in the **Mil-**scheme, where the sign of the $\frac{1}{2}gg^{\prime }$ term is changed in the corrector step, a modification made in the spirit of the derivation of Equation (10). Note, however, that the Heun scheme has a strong convergence O(h), contrary to the claim of [9]: a similar result was found in [18].

On the other hand, the strong convergence found for the higher-order Taylor-based schemes is rather surprising. The **T3/2** algorithm shows strong convergence of only O(h), despite being based on a Taylor expansion that includes terms up to $O(h^{3/2})$. This issue is even more acute for the **CUP1** and **CUP2** schemes, which also exhibit strong convergence of only O(h), despite their Taylor expansion (Equation (13)), including terms up to $O(h^{2})$. We also examined the Runge–Kutta scheme of [23], which includes terms $O(h^{2})$ and was believed to have a strong convergence $O(h^{3/2})$, and found its strong convergence to be only O(h). To rule out possible numerical artifacts or coding errors, we repeated the assessment of the Taylor-based schemes using SDE dx=xdt+Dx∘dW, which can be solved analytically: it was possible to confirm that the integration routines were integrating correctly and the overall numerical approach was sound.

### 4.2. Stability

To establish the parameter regions where the integration remains stable, the system was simulated by varying the parameters *D* and *h*: 20 values for *D* and 20 values for *h* were selected, as shown in Figure 2. For all simulations and algorithms, $3\times 10^{4}$ trajectories, each starting from a random point drawn from the equilibrium distribution, were tracked up to a time of $10^{4}$. Thus, if $h=10^{-2}$, each trajectory was integrated for $10^{4}/h=10^{6}$ elementary time steps. If |x|>100 at any point during the integration for any of the trajectories, we marked that parameter set as unstable for the given algorithm.

The results of these simulations are shown in Figure 2. The “Stability” column in Table 1 reports the number of (D,h) parameter pairs for which the simulations remained stable (i.e., did not diverge) for each algorithm.

The **Heun** scheme proved to be the most stable, followed by **HeSt**, **HePC**, and **Mil-**. Conversely, the Taylor-based schemes performed worse, particularly **CUP1** and **CUP2**. This result is not surprising, as the introduction of a corrector step in a numerical integration scheme is known to improve stability.

### 4.3. Stationary Distributions

To study the stationary distributions, we performed simulations across a grid of parameters, using 20 values for *h* in the range [0.01, 0.2] and 10 values for *D* in the range [0.01, 0.25]. For each integration scheme and for each (D,h) pair, we computed $4\times 10^{5}$ trajectories with initial conditions drawn from the Stratonovich theoretical equilibrium distribution. Each trajectory was integrated up to a time $T=10^{4}$ and sampled at intervals of ΔT=1.0. The value of *x* at each sample was accumulated to construct the equilibrium probability density, hereafter denoted as P(x). In total, each P(x) is constructed from $4\times 10^{9}$ data points.

**Figure 3:**
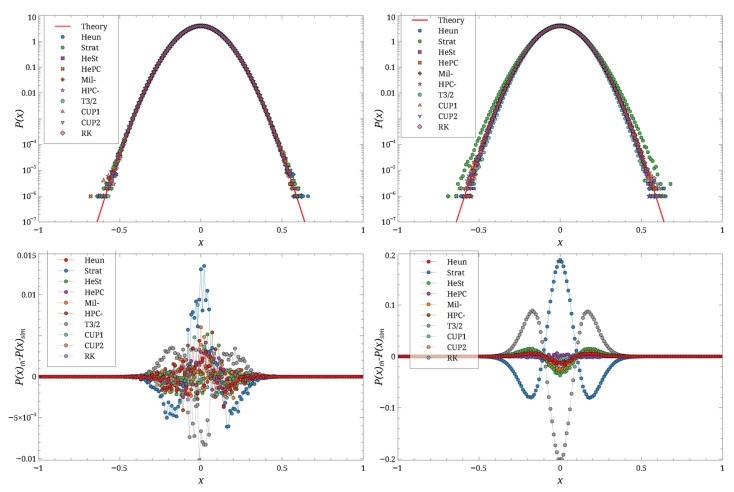
Simulation results, carried out for $D=0.1\times 10^{-1}$, h=0.01 (**left**), and h=0.2 (**right**). Top row shows P(x), bottom row $P_{eq}\left(x\right)-P\left(x\right)$. Different symbols are the different schemes.

**Figure 4:**
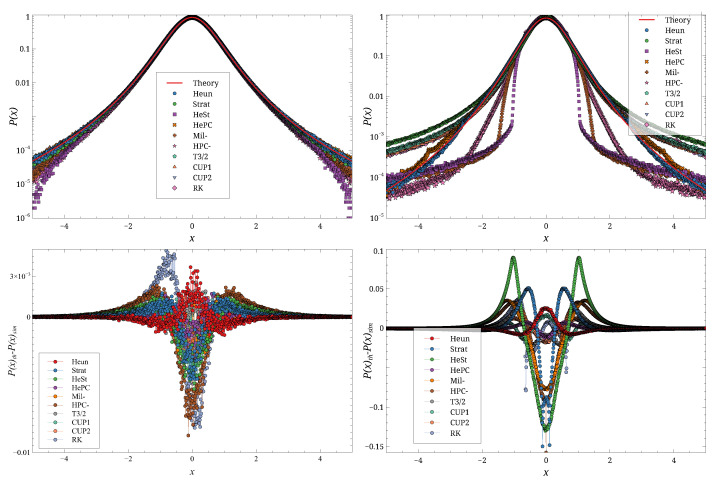
Simulation results, carried out for $D=2.5\times 10^{-1}$, h=0.01 (**left**), and h=0.2 (**right**). Top row shows P(x), bottom row $P_{eq}\left(x\right)-P\left(x\right)$. Different symbols are the different schemes.

Typical results are shown in Figure 3 for a small *D* value, in Figure 4 for a large *D* value, and for two different values of *h*.

These figures show that for small *h*, the agreement between theory and simulation is good for both values of *D*. As *h* increases, the agreement deteriorates, with the magnitude of the deviation depending on the integration scheme. For instance, the results for D=0.25 and h=0.2 (Figure 4, right) show the emergence of spurious structures for some schemes. Overall, the **Stra** scheme is the first to deviate from the theoretical distribution. At h=0.2, the **HePC** scheme appears to yield the distribution closest to the theory, followed by **CUP2**, **CUP1**, and **Heun**, for both values of *D*.

To quantitatively assess the deviation of the simulated distribution P(x) from the theoretical one $P_{eq}\left(x\right)$, we introduced two metrics. The first, which we term the “distance,” is defined as $\int |P_{eq}\left(x\right)-P\left(x\right)|dx$. The second, the “ratio,” is defined as $\int |\left(P_{eq}\left(x\right)-P\left(x\right)\right)/P_{eq}\left(x\right)|dx=\int |1-P\left(x\right)/P_{eq}\left(x\right)|dx$, where this integral is restricted to the region where $P_{eq}\left(x\right)\geq 10^{-4}P_{eq}\left(0\right)$. The distance metric emphasizes discrepancies near the origin where the probability density is large and statistics are robust, but it is less sensitive to differences in the tails. Conversely, the ratio metric places more weight on the tails, a region where statistics may be less reliable. For both metrics, a smaller value indicates better agreement between the simulated and theoretical distributions.

**Figure 5:**
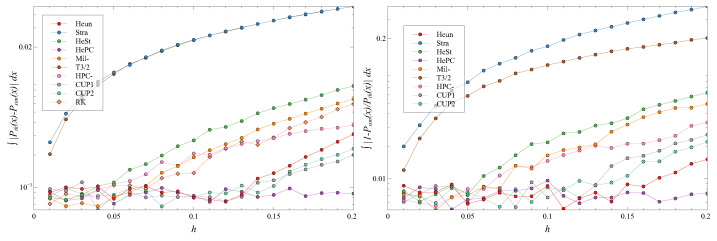
The distance $\int |P_{eq}\left(x\right)-P\left(x\right)|dx$ (**left**) and the ratio $\int |1-P\left(x\right)/P_{eq}\left(x\right)|dx$ (**right**) for different *h*s and D=0.01. Note the log scale on the ordinates. Different symbols are the different schemes.

**Figure 6:**
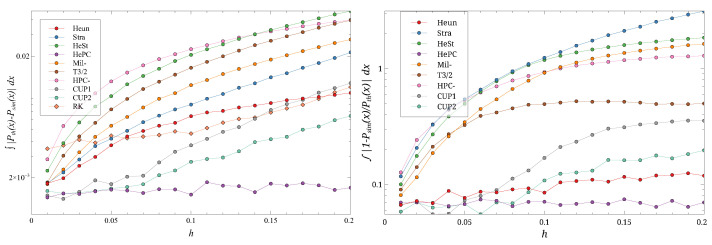
The distance $\int |P_{eq}\left(x\right)-P\left(x\right)|dx$ (**left**) and the ratio $\int |1-P\left(x\right)/P_{eq}\left(x\right)|dx$ (**right**) for different *h*s and D=0.09. Note the log scale on the ordinates. Different symbols are the different schemes.

**Figure 7:**
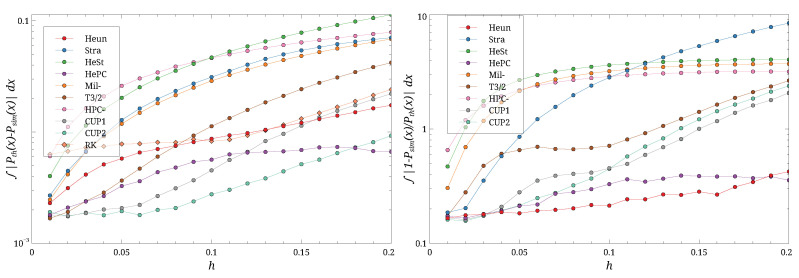
The distance $\int |P_{eq}\left(x\right)-P\left(x\right)|dx$ (**left**) and the ratio $\int |1-P\left(x\right)/P_{eq}\left(x\right)|dx$ (**right**) for different *h*s and D=0.17. Note the log scale on the ordinates. Different symbols are the different schemes.

**Figure 8:**
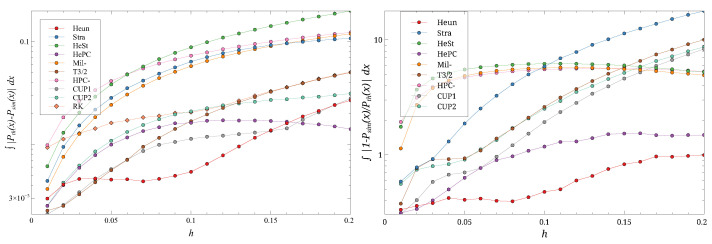
The distance $\int |P_{eq}\left(x\right)-P\left(x\right)|dx$ (**left**) and the ratio $\int |1-P\left(x\right)/P_{eq}\left(x\right)|dx$ (**right**) for different *h*s and D=0.25. Note the log scale on the ordinates. Different symbols are the different schemes.

Figure 5, Figure 6, Figure 7 and Figure 8 show the behavior of the two metrics as *h* is increased, for four *D* values.

As expected, both the distance and ratio metrics typically increase with *h*. For the smallest value of *D* considered (Figure 5), the distance between simulation and theory is small for most schemes at small *h*, with the **Stra** and **T3/2** schemes being notable exceptions. As *h* increases, the distance for the **HePC** scheme remains roughly constant, while it grows for the others. At the largest *h* considered, **HePC** performs best, followed by **CUP1**, **CUP2**, and **Heun**. The behavior of the ratio metric is similar, with only minor changes in the relative performance of **CUP1**, **CUP2**, and **Heun** at large *h*.

The evolution of the metrics for the largest *D* considered (Figure 8) shows some interesting differences. At small *h*, the **HePC** scheme no longer stands out. It emerges as the best performer in terms of distance only as *h* increases, and its advantage is less pronounced than for smaller *D*. In terms of the ratio, the **Heun** scheme performs best, followed by **HePC**.

The results for intermediate values of *D*, shown in Figure 6 and Figure 7, exhibit transitional behavior between these two extremes for both metrics.

## 5. Conclusions

In this work, we have conducted a critical re-examination of the Heun algorithm and several of its variants for the numerical integration of Stochastic Differential Equations. Motivated by the fact that the standard Heun scheme is based on the low-order Euler method, we systematically evaluated a dozen different algorithms, assessing their performance based on three key criteria: strong convergence, numerical stability, and the ability to reproduce correct long-time equilibrium distributions.

Our extensive numerical simulations lead to several key conclusions. First, despite its simplicity, the standard **Heun** scheme demonstrates remarkable robustness. It was found to be the most stable of all tested algorithms across a wide range of parameters and consistently produced accurate equilibrium distributions, especially when considering metrics that weight the tails of the distribution. This confirms its status as a reliable benchmark for SDE integration. There are some rare cases when **HePC** fared better, but given that this scheme requires several evaluations of the quantities f(x) and g(x) it should be weighted carefully whether to prefer **HePC** to an integration carried out with **Heun** and a smaller *h*.

Second, a surprising and significant finding is the underperformance of theoretically higher-order schemes. Taylor-based schemes, such as **T3/2** and **CUP**, which include terms up to $O(h^{3/2})$ and $O(h^{2})$, respectively, failed to achieve a strong convergence order beyond O(h). This suggests that simply including more terms from a Taylor expansion does not guarantee improved strong convergence in practice and highlights the complex relationship between theoretical order and numerical performance. These higher-order schemes also proved to be significantly less stable than the predictor-corrector type methods.

Third, our analysis of strong convergence has clarified a point of confusion in the literature. We have shown conclusively that the Heun scheme has a strong convergence of order O(h), disproving an earlier claim that suggested an order of $O(h^{3/2})$.

## Data Availability

Data are available upon reasonable request. F90 codes are available at https://github.com/dundacil/heun_extension, accessed on 10 August 2025.
